# Acoustically Driven Hybrid Nanocrystals for In Vivo Pancreatic Cancer Treatment

**DOI:** 10.1021/acsami.4c21975

**Published:** 2025-02-17

**Authors:** Marzia Conte, Marco Carofiglio, Robin Shae Vander Pol, Anthony Wood, Nathanael Hernandez, Ashley Joubert, Camden Caffey, Corrine Ying Xuan Chua, Alessandro Grattoni, Valentina Cauda

**Affiliations:** † Department of Applied Science and Technology, Politecnico di Torino, Corso Duca degli Abruzzi 24, 10129 Turin, Italy; ‡ Department of Nanomedicine, Houston Methodist Research Institute, Houston, Texas 77030, United States; § Department of Surgery, Houston Methodist Research Institute, Houston, Texas 77030, United States; ∥ Department of Radiation Oncology, Houston Methodist Research Institute, Houston, Texas 77030, United States

**Keywords:** pancreatic cancer, zinc oxide NPs, sonodynamic therapy, IR780 sonosensitizer, ultrasound, immune cells, in vivo models

## Abstract

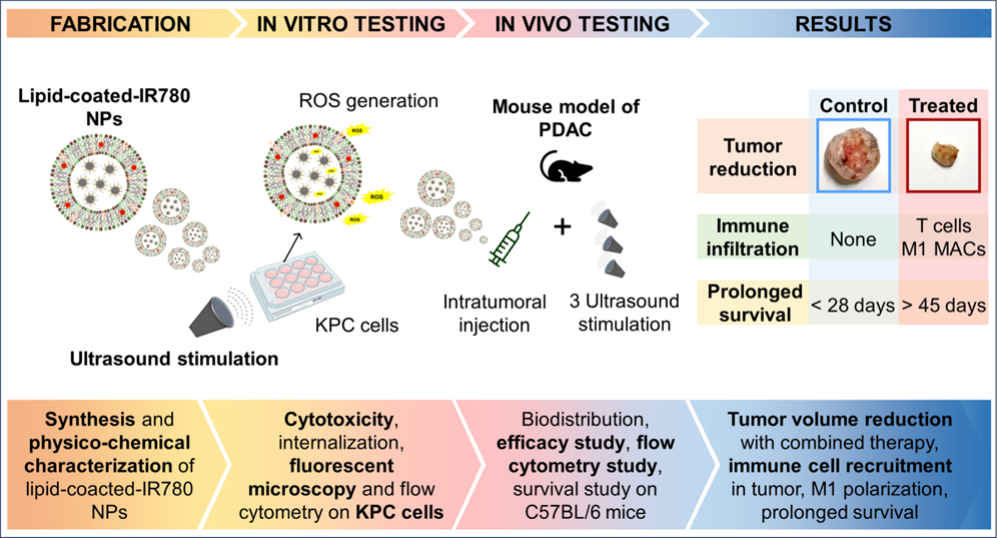

New treatment strategies are urgently needed for pancreatic ductal adenocarcinoma (PDAC), which is one of the deadliest tumors nowadays. PDAC is marked by hypoxia, intrinsic chemoresistance, a “cold” tumor microenvironment, and dense desmoplastic stroma, which hinders drug penetration. This study investigates the combined effect of iron-doped, lipid-coated zinc oxide nanoparticles enhanced with a fluorescent sonosensitizer and local ultrasound stimulation in treating PDAC. Nanoparticles were synthesized and coated by lipids, and their physiochemical properties were characterized by assessing reproducibility, stability, and efficient inclusion of the sonosensitizer. In vitro, sonosensitizer-enhanced nanoconstructs were tested on a KPC murine PDAC cell line in combination with ultrasound to evaluate their cytotoxicity and assess their efficacy. In vivo, NPs were further coupled with AlexaFluor 700 to allow their localization over time, and the nanoconstructs were intratumorally administered to a subcutaneous murine PDAC model to enhance local bioavailability and tumor visualization and minimize off-target effects of systemic delivery. Biodistribution, efficacy, flow cytometry, and survival studies were carried out on different cohorts of mice. The sonosensitizer-enhanced nanoconstructs, combined with ultrasound, triggered significant reactive oxygen species (ROS) production, reducing the KPC cell viability. In vivo, the antitumor efficacy was particularly pronounced with ultrasound stimulation, demonstrating a synergistic interaction between the nanoparticles and ultrasound. Moreover, increased immune cell infiltration, enhanced cancer cell apoptosis, and prolonged survival of the treated animals were achieved. These findings highlight the potential of a synergistic therapeutic approach combining lipid-coated sonosensitizer-loaded nanoparticles and ultrasound stimulation as an effective therapy for PDAC and in situ monitoring.

## Introduction

Pancreatic ductal adenocarcinoma (PDAC) remains one of the most difficult cancers to treat. Its “cold” tumor immune microenvironment, dense desmoplastic stroma, and poorly organized vasculature causing hypoxia impede immune cell recruitment.^1,2^ Innovative strategies are urgently needed to overcome this challenge. Current research focuses on multimodal approaches for cancer treatment, where different therapies synergistically lead to more successful tumor elimination than single treatments.^3^ However, deep-seated organs like the pancreas are difficult to reach, making locally applied therapies like photodynamic therapy (PDT), photothermal therapy (PTT), radiotherapy, and radiodynamic therapy (RDT) less effective^4,5^ and often associated with severe side effects.^6,7^ Conversely, sonodynamic therapy (SDT) offers a promising balance between low side effects, deep tissue penetration, and exceptional spatiotemporal selectivity.^8,9^ SDT’s cytotoxic mechanism is based on inertial cavitation, where gas bubbles violently collapse generating reactive oxygen species (ROS) that induce cancer cell apoptosis.^10,11^ Ultrasound treatments applied as cancer therapy have been proven to elicit an inflammatory immune activation, not only at the application site but also systemically.^9^

The release of tumor cell debris and apoptotic bodies/necrosis fragments has the potential to elicit the so-called immunogenic cell death (ICD),^12^ a mechanism that activates damage-associated molecular patterns (DAMPs),^13^ leading to dendritic cell maturation and migration to lymph nodes. Here, they present tumor antigens to CD4^+^ and CD8^+^ T cells, which in turn are activated and redirected to systemic circulation to finally infiltrate the tumor mass.^14^

PDAC therapies do not rely on the tumor’s enhanced permeability and retention effect; transcytosis rather than passive accumulation is the main route for drug or nanoconstruct infiltration in the tumor.^15^ Ultrasounds may enhance tumor permeability and vessel fenestration, improving tumor accumulation in both intratumoral and intravenous administrations.^16,17^ However, SDT alone can be impaired by PDAC’s hypoxic nature, since the lack of available oxygen severely reduces the cavitation effect and consequently ROS generation, requiring smart nanomedicine-based strategies.^8,17−22^

This study proposes a multimodal therapy using lipid-coated iron-doped zinc oxide (ZnO) nanoparticles (NPs) stimulated by a clinically approved ultrasound transducer. ZnO NPs have inherent anticancer properties, with a dose-dependent toxicity relying on various mechanisms.^23^ To enhance the biocompatibility of ZnO NPs and ensure that they remain intrinsically safe unless activated via ultrasound, iron is incorporated as a doping agent. This incorporation leads to significant improvements in their physicochemical properties, including a slower dissolution rate and a more favorable toxicity profile. These enhancements, extensively documented in the literature,^24,25^ were also thoroughly detailed in a previous study from our research group,^26^ establishing iron-doped ZnO NPs as an ideal starting biocompatible material for further optimization. Functionalizing ZnO with amino-propyl groups allows gas nanobubbles absorption, enhancing inertial cavitation even in hypoxic conditions.^27^ A tailored lipidic shell inspired by COVID-19 vaccines boosts tolerability and prevents aggregation,^28^ while incorporating the lipophilic sonosensitizer IR780 maximizes ultrasound stimulation effects and offers imaging potential, due to its strong optical absorption in the near-infrared spectrum.^29−32^

To date, only a few research groups have explored similar treatment combinations using nanoparticles-assisted ultrasound.^33−36^ This prompted a focus on a proof-of-concept study of the multimodal therapy starting with intratumoral administration of the NPs. This approach was chosen due to the poor blood perfusion considered a hallmark of PDAC.^3,37^ Intratumoral drug administration would help concentrate the nanoconstructs in the tumor area and further deeply distribute them under the effect of US stimulation. Additionally, local administrations have been shown to mitigate side effects compared to systemic administration.^38^ In vitro tests further suggested that the tumor cell cytotoxicity is dose-dependent,^39−41^ leading to the search for approaches that improve their retention in the tumor area within the first 48 h post-administration, without contending with barriers like the dense extracellular matrix and high interstitial fluid pressure, often seen in PDACs.

Here, the aim was to demonstrate that intratumoral injection of these lipid-coated ZnO NPs followed by ultrasound (US) stimulation could reduce tumor burdens and elicit both local and systemic responses. The nanoconstructs were tested in vitro for cytotoxicity, internalization, and mechanisms of action and then validated in vivo for biodistribution, side effects, and efficacy in the presence or absence of the US treatment. In vivo studies confirmed that both the nanoconstructs and ultrasound application, when administered individually, were well tolerated, with no observed toxicity issues. Combined, they caused a synergistic cytotoxic response, resulting in tumor shrinkage with tumor cell apoptosis and immune cells recruitment. The mice treated with the lipid-coated NPs or lipid-coated-IR780 NPs in combination with US showed significantly prolonged survival compared to that of controls, demonstrating the efficacy of the combined treatment.

## Results and Discussion

### Fabrication and Characterization of Lipid-Coated ZnO NPs Enhanced with IR780

**Figure 1:**
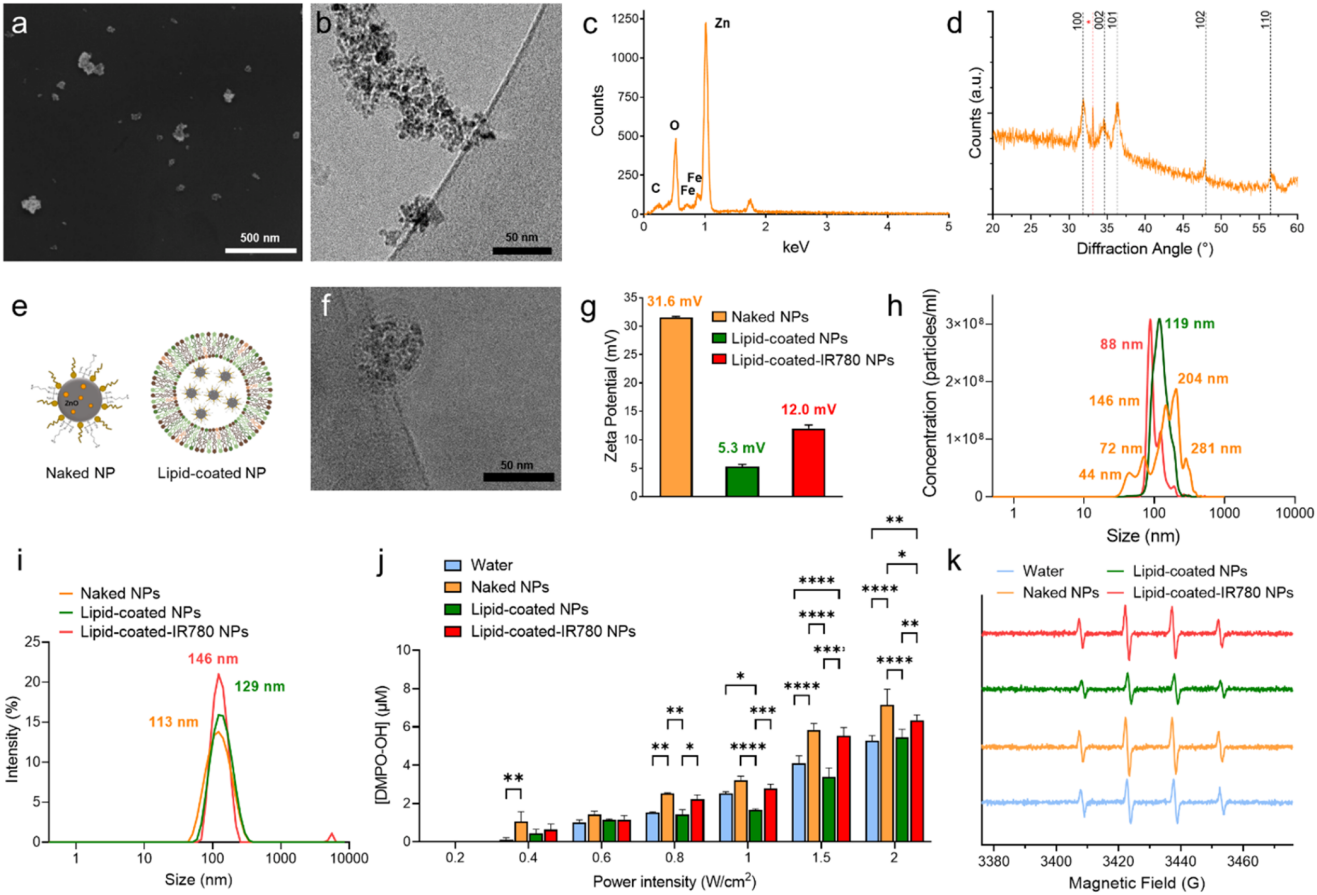
(a) SEM picture, (b) CryoEM image, (c) EDX, and (d) XRD of the naked NPs. (e) Schematic representation of naked and lipid-coated NPs. (f) CryoEM image of lipid-coated NPs. (g) ζ-Potential and (h) NTA of naked (orange), lipid-coated (green), and lipid-coated-IR780 (red) NPs. (i) DLS of the naked (orange), lipid-coated (green), and lipid-coated-IR780 (red) NPs. (j) EPR measurements of ROS production of the various nanoconstructs in water upon ultrasound stimulation. (k) Representative image of the spin-adduct of the DMPO-OH complex obtained after US stimulation at 1.5 W/cm^2^. Data are expressed as mean ± standard deviation. Significance was analyzed by two-way ANOVA. *p < 0.05; **p < 0.005; ***p < 0.0005; ****p < 0.0001. Tukey’s correction was applied for multiple comparison.

The iron-doped ZnO NPs were obtained following an established wet-chemical synthesis protocol.^26^ They had a diameter of about 8–10 nm, as confirmed by the scanning electron microscopy (SEM) images (Figure 1a), and tended to form irregular aggregates in aqueous media, as observed in cryoelectron microscopy (CryoEM) images (Figure 1b). Energy-dispersive X-ray (EDX) analysis confirmed a distinct peak for zinc and the presence of iron doping, with a final atomic ratio of 3.55 atom % (Figure 1c). X-ray diffraction (XRD) pattern displayed diffraction peaks typical of the wurtzitic structure of ZnO (JCPDS-ICDD, Card No. 89-1397), with no additional peaks indicating other oxide phases from unwanted iron nucleation (Figure 1d). A lipidic shell, inspired by COVID-19 vaccines and composed of a mixture of negatively charged, neutral, PEGylated lipids and cholesterol was used to coat the ZnO NPs via a straightforward solvent exchange method.^28^

When coated with the lipidic shell (Figure 1e), the final nanoconstructs had a size of approximately 50 nm, as confirmed by CryoEM imaging, depicting the lipid bilayer incorporating multiple NPs (Figure 1f). Zinc release from naked or lipid-coated NPs in saline or cell culture media over time was measured using inductively coupled plasma-optical emission spectrometry (ICP-OES, Figure S1a,b). Less than 20% of total zinc was released into the supernatant after 1 week for all groups. Naked NPs tended to dissolve more easily in cell culture medium than in saline, likely due to phosphate groups forming complexes with zinc and increasing the solubility. In contrast, lipid-coated NPs showed similar degradation in both water and medium, indicating higher stability in different aqueous environments (Figure S1c).

To potentiate the effects of US stimulation, IR780 sonosensitizer was added to the lipidic coating of NPs exploiting its lipophilic nature and ensuring adequate retention of the dye in the shell (Figure S2a–d).

Compared to pristine NPs, lipid-coated NPs exhibited a marked shift in ζ-potential, likely due to the interaction between the positively charged ZnO NPs and the negatively charged lipidic shell. In the presence of IR780, a cationic lipophilic dye, the ζ-potential consequently increased slightly (Figure 1g).^28^ Nanoparticle tracking analysis (NTA) showed naked NPs forming clusters of various sizes (orange line), while lipid-coated NPs displayed a monodisperse size distribution (single green peak). Therefore, coating of the NPs improved their dispersion in aqueous media, overcoming the tendency of pristine inorganic NPs such as ZnO to aggregate^42,43^ in view of in vivo applications. The incorporation of IR780 did not affect much the monodispersity of the nanoconstructs in aqueous medium (Figure 1h,i).

Electron Paramagnetic Resonance (EPR) spectroscopy indicated that the NPs enhanced the ROS production in water upon US stimulation. While pure water could generate ROS through inertial cavitation induced by US, ZnO NPs reduced the cavitation threshold and enhanced ROS concentration.^44^ Iron-doped ZnO NPs showed increased ROS production at US power densities of 0.4, 0.8, 1.5, and 2 W/cm^2^, compared to water. In contrast, lipid-coated NPs produced less ROS suggesting lipids may scavenge the hydroxyl radicals’ production. Despite this, lipidic coating was necessary to safely administer the ZnO NPs due to their inherent cytotoxicity at high dosages and to reduce their aggregation. Thus, the inclusion of the sonosensitizer IR780 played a pivotal role in the nanoconstructs’ effectiveness by restoring ROS production. Indeed, lipid-coated-IR780 NPs produced comparable ROS to naked NPs at power densities up to 1.5 W/cm^2^ (Figure 1j,k). These results indicated promising potential for subsequent in vitro and in vivo efficacy studies, as the lipidic shell ensures high biocompatibility and colloidal stability, while ZnO NPs and IR780 enhance the response to US stimulation.

Lipid-coated NPs were dispersed in water at 1 mg/mL, which is suitable for in vitro applications.^28^ For in vivo applications, lipid-coated NPs were concentrated in 0.9% w/v saline (Figure S2e). The ζ-potential and DLS showed surface charge (+4.0 mV) and dispersibility in saline solution comparable to those in water (Figure S2f). Concentrations of up to 40 mg/mL were thus achieved for in vivo intratumoral injections. The incorporation of fluorescent dyes, necessary to visualize NPs in subsequent in vitro and in vivo experiments, did not affect the dispersibility or the lipidic coating of the NPs (Figure S2h,i).

### In Vitro Validation of the Combined NPs/US Treatment on KPC Cells

**Figure 2:**
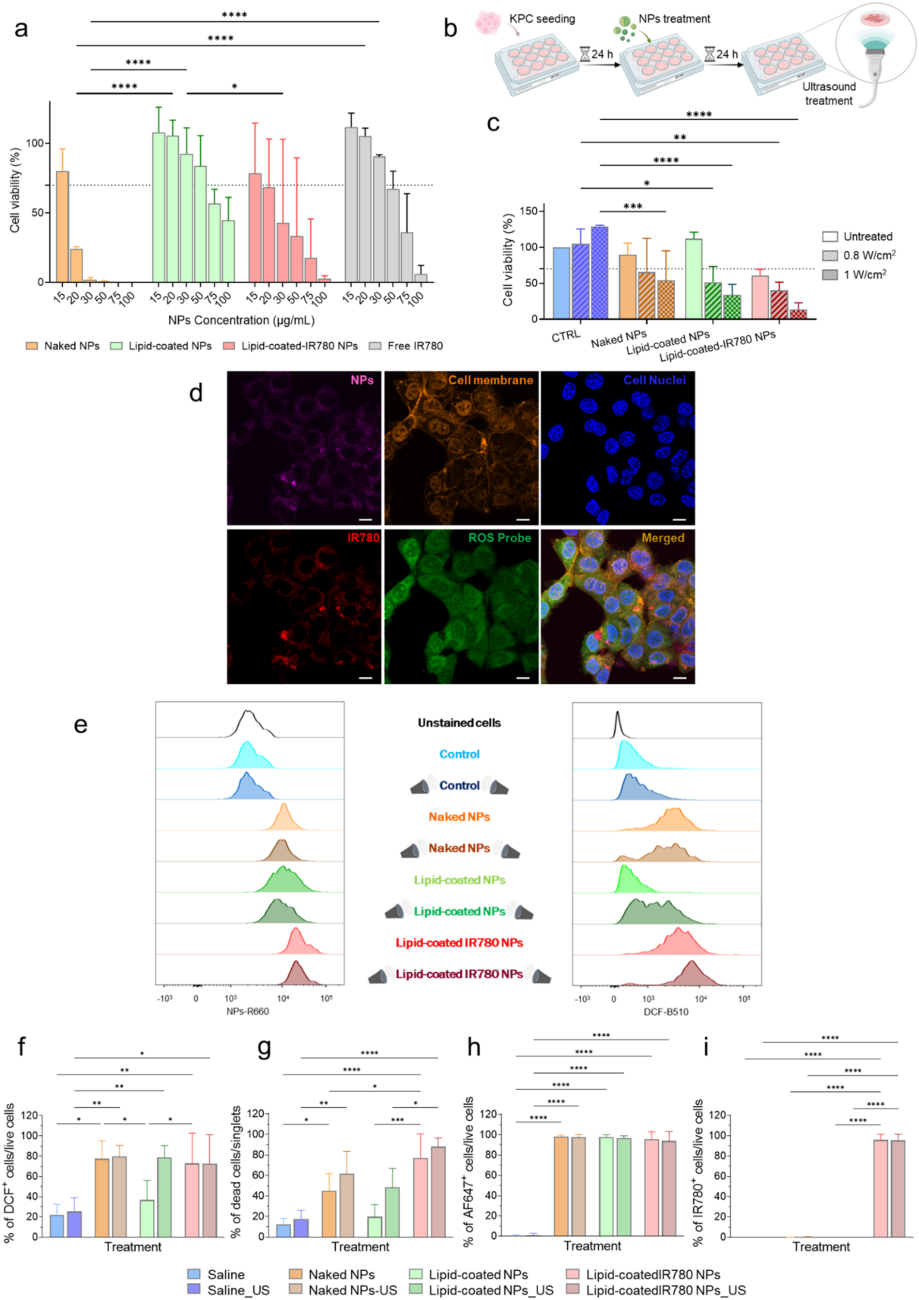
(a) Cytotoxicity of naked NPs, lipid-coated NPs, lipid-coated IR780 NPs, and free IR780 on KPC cells after 24 h exposure. (b) Schematic protocol of in vitro tests. (c) Cell viability of KPC cells after NPs administration, with and without US stimulation, measured 24 h after US. (d) Fluorescence confocal microscopy of KPC cells internalizing lipid-coated-IR780 NPs after US stimulation at 0.8 W/cm^2^ for 1 min. The NPs, labeled with AlexaFluor 647, are shown in the pink channel, IR780 is imaged in the red channel, nuclei are stained with DAPI (blue channel), membranes with WGA 550 (orange channel), and the green channel shows DCF signal due to the generation of ROS. (e) One-dimensional histograms of the fluorescence intensity associated with NPs internalization (NPs-R660) and DCF production (DCF-B510) for all treatment groups. (f) Histograms reporting the percentage of DCF-producing cells, (g) dead cells, (h) cells internalizing NPs, and (i) cells internalizing IR780 (US treatment 0.8 W/cm^2^, 1 min, 100% DC). Data are expressed as mean ± standard deviation. Significance was analyzed by two-way ANOVA. *p < 0.05; **p < 0.005; ***p < 0.0005; ****p < 0.0001. Tukey’s correction was applied for multiple comparison.

Naked, lipid-coated, and lipid-coated-IR780 NPs were tested on KPC cells at increasing concentrations to assess their cytotoxicity after 24, 48, and 72 h exposure (Figures 2a and S3a,b, respectively). At a concentration of 20 μg/mL, naked NPs markedly affected cell viability after 24 h of exposure (Figure 2a). Following a threshold of 70%,^45^ lipid-coated NPs were noncytotoxic up to 50 μg/mL owing to the presence of the lipidic shell, as also previously reported.^28^ When IR780 was incorporated into the lipidic shell, there was a decrease in cell viability, while the free IR780 counterpart exhibited no significant toxicity up to 50 μg/mL.

To compare all treatment groups, an NPs concentration of 15 μg/mL was chosen to perform the following in vitro experiments. The viability of KPC cells was assessed after 24 h post US treatment at 0.8 and 1 W/cm^2^ following NPs administration (Figure 2b). Control KPC cells (without NPs treatment) were unaffected by US stimulation, and US stimulation at both doses did not significantly affect KPC cells also treated with the naked NPs. Strikingly, US stimulation significantly decreased cell viability with lipid-coated NPs, in a power density-dependent manner, with a *p*-value of <0.0001 at 1 W/cm^2^ compared to control US stimulation alone. Comparatively, the incorporation of IR780 further enhanced the impact of lipid-coated NPs on the cell viability. In particular, upon US exposure at 1 W/cm^2^, the recorded cell viability with lipid-coated-IR780 was significantly lower (*p* < 0.0001) than with control US administration, and markedly lower than the cytotoxicity produced by lipid-coated NPs and US (Figure 2c).

Confocal microscopy imaging showed the KPC cell internalization of lipid-coated IR780 NPs after US stimulation at 0.8 W/cm^2^. Specifically, the NPs (labeled with AlexaFluor 647) and IR780 dye were visible in the cytoplasm. Further, ROS production was detected via the 2′,7′-Dichlorofluorescin (DCF) signal, which showed cytosolic presence (Figure 2d).

Flow cytometry was employed to assess the fluorescence intensity associated with internalized NPs (NPs-R660) and ROS generation (DCF-B510) among various treatment groups (Figures 2e and S3c). Quantitative assessment of the percentage of cells expressing the DCF signal showed that ROS production was increased in all NPs-treated groups, with and without US stimulation (0.8 W/cm^2^), compared to that of control cells. Remarkably, the group treated with lipid-coated NPs alone did not cause excess of ROS generation or enhanced cell death. However, when the reaction was stimulated with US, an increase in ROS production was observed (Figure 2f). These results strongly suggest that the lipidic shell minimized zinc oxide NPs toxicity and reduced oxidative stress, as further confirmed by the low percentage of dead cells in this treatment group, comparable to that of untreated controls (Figure 2g). Indeed, only the combination with US restored ROS production and cell death to the levels observed in other treatment groups. The percentage of cells internalizing NPs and IR780 was over 95% for all treated groups, proving that the production of ROS is effectively intracellular (Figure 2h,i, respectively).

Thus, in conclusion to the in vitro experiments, we can assume that the lipid-coated-IR780 NPs are more cytotoxic across all concentrations compared to lipid-coated NPs. Furthermore, US increases the cytotoxicity of lipid-coated IR780 NPs, although the production of ROS in KPC cells remains comparable. Although preliminary data (Figure 1i) showed a significant improvement of ROS production, it is important to note that these experiments were conducted in a solution volume of NPs (see the Experimental Section). In contrast, the flat cell monolayer considered in 2D experiments may not be ideal for fully unraveling the mechanisms of the NPs + US combination. Ultrasounds consist of acoustic pressure waves propagating in a volume (of liquid or tissue) and likely have minimal effect on a single-cell surface, such as the in vitro monolayer. Therefore, in vivo experiments are needed to better understand the phenomenon in a three-dimensional tumor model and to demonstrate therapeutic efficacy.

### Biodistribution Study of NPs Injected in Murine Models of PDAC

**Figure 3:**
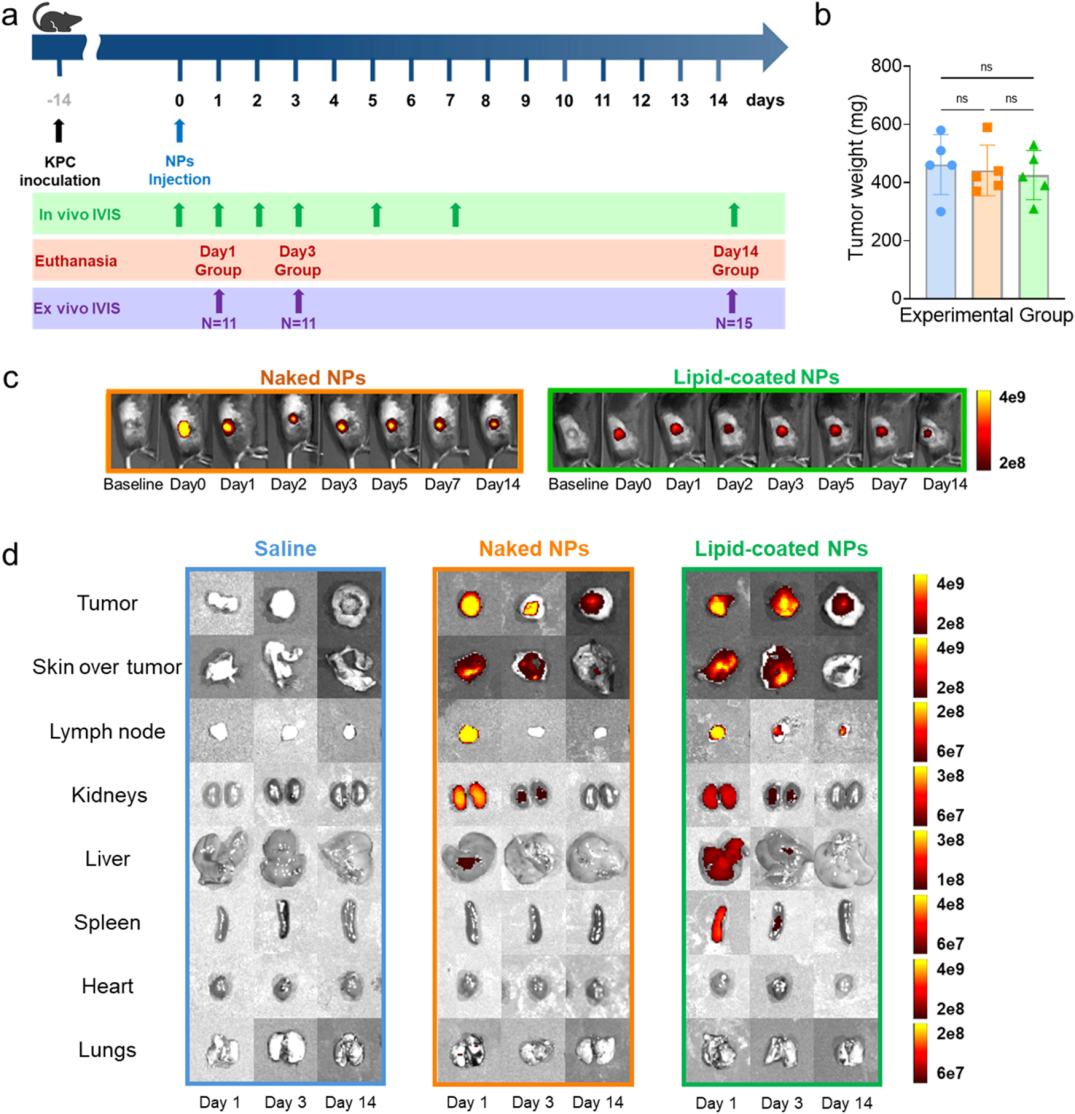
(a) Scheme of the treatment plan. (b) Tumor weights on day 14. (c) In vivo IVIS imaging over time of naked and lipid-coated NPs dyed with AlexaFluor 700 (excitation 675 nm, emission 720 nm). (d) IVIS pictures of organs explanted at various time points (excitation 675 nm, emission 720 nm). Data are expressed as mean ± standard deviation. Significance was analyzed by either one-way or two-way ANOVA. *p < 0.05; **p < 0.005; ***p < 0.0005; ****p < 0.0001. Tukey’s correction was applied for multiple comparison.

A biodistribution study was performed by injecting the naked NPs and lipid-coated NPs intratumorally into a KPC subcutaneous murine model of PDAC. Mice receiving saline only were used as controls. The mice were imaged with IVIS pre- and post-NPs administration (day 0) and at predetermined time points. Ex vivo IVIS was performed on days 1 (*n* = 11), 3 (*n* = 11) and 14 (*n* = 15) (Figure 3a). Tumor weights measured ex vivo were comparable across treatment groups, indicating no significant effects of NPs on tumor shrinkage (Figure 3b). This finding was corroborated by the in vivo progression of tumor volumes (Figure S4a) and visual comparisons of extracted tumors, which showed no differences among experimental groups (Figure S4b). The mice exhibited no signs of toxicity, as evidenced by stable body weight (Figure S4c) and basal temperature (Figure S4d), further confirming the safety of NPs injection. Throughout the study, the fluorescent signal of both naked and lipid-coated NPs (labeled with AlexaFluor 700 covalently conjugated to ZnO NPs) remained clearly detectable (Figure 3c). The fluorescence intensity of lipid-coated NPs was consistently lower than that of the naked NPs. This could be due either to the lipidic coating’s shielding and quenching effect or the improved dispersibility of NPs when surrounded by the lipid bilayer.^46^ Initial systemic accumulation in filtration organs (lymph nodes, kidneys, and spleen) was observed in ex vivo IVIS images on day 1 and to a lesser extent on day 3. Both naked and lipid-coated NPs were rapidly expelled, with no detectable traces in organs other than tumors and surrounding skin by the end point day 14 (Figure 3d). Tumors extracted on days 1, 3, and 14 were analyzed with the ICP-OES to detect residual zinc, expressed as a percentage of the initially injected dose (% ID). A declining trend in zinc retention was observed, with up to 80% of naked NPs retained in the tumors on day 1, decreasing to 20% of the injected dose by day 14 (Figure S5a). Tumors injected with lipid-coated NPs exhibited a lower initial zinc accumulation, following a similar descending trend over time, which, however, was not statistically significant. On the other hand, all plasma samples showed zinc levels around 1% of the injected dose, similar to saline controls, suggesting the absence of systemic zinc diffusion (Figure S5b).

### Efficacy Study of the Combined NPs/US Treatment

**Figure 4:**
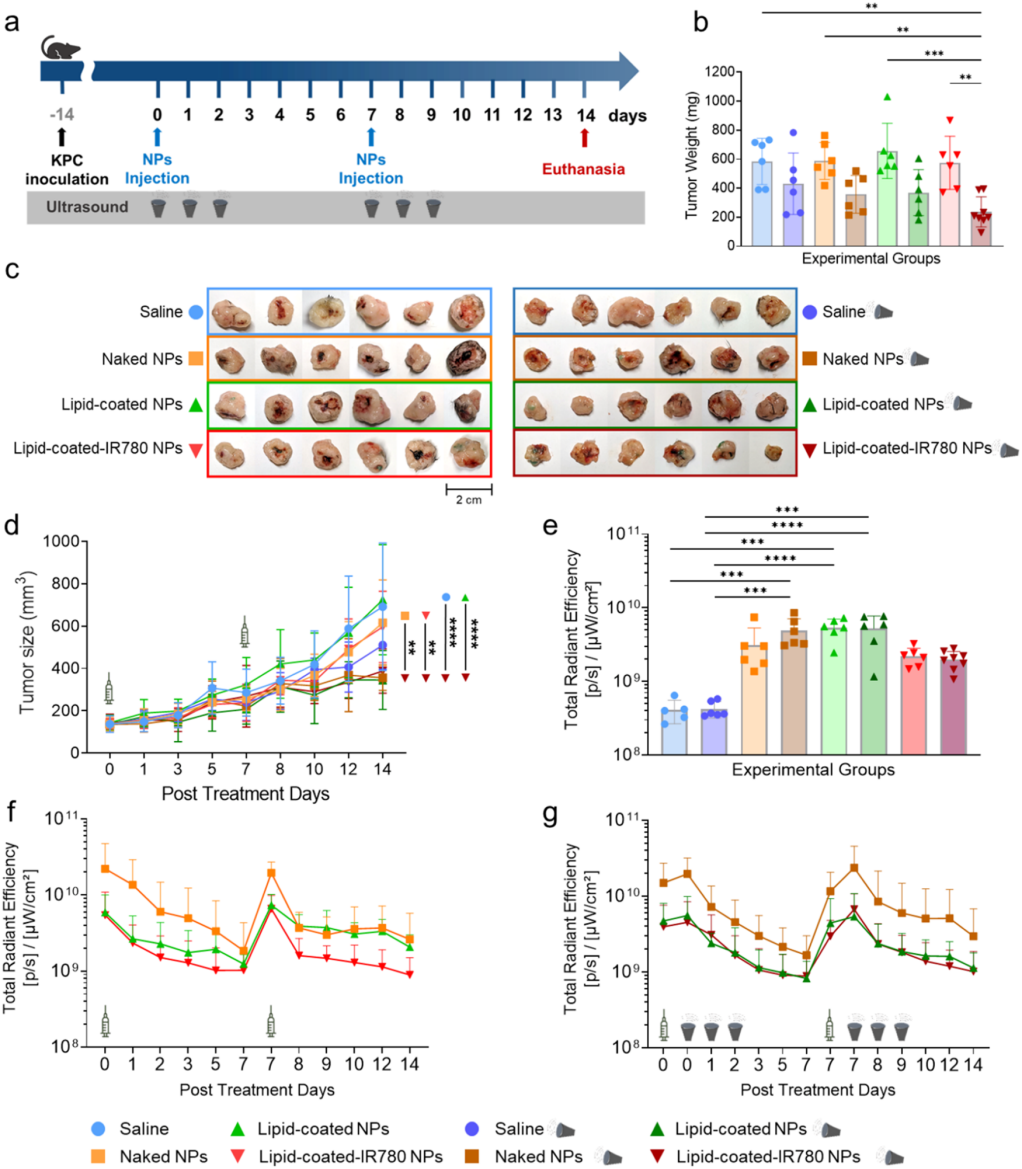
(a) Scheme of the treatment plan. (b) Tumor weights on day 14. (c) Digital photos of tumors explanted on day 14. (d) Tumor volume progression in vivo over time. (e) Total radiant efficiency (excitation 675 nm, emission 720 nm) of the explanted tumors. (f) In vivo total radiant efficiency progression within tumor regions of interest (ROIs) of the groups treated with NPs only and (g) those treated with NPs and ultrasound (excitation 675 nm, emission 720 nm). Data are expressed as mean ± standard deviation. Significance was analyzed by either one-way or two-way ANOVA. *p < 0.05; **p < 0.005; ***p < 0.0005; ****p < 0.0001. Tukey’s correction was applied for multiple comparison.

The biodistribution study results showed that intratumoral injection of the nanoconstructs was safe per se and did not elicit any adverse reactions. Therefore, an efficacy study was carried out by combining intratumoral injection of the NPs and US stimulation, including a treatment group with the sonosensitizer. IVIS imaging data revealed a robust fluorescent signal persisting up to 48 h postinjection. This finding supported the strategy of applying US stimulation after NPs administration daily over 3 consecutive days to ensure their tumor retention during the treatment. Additionally, a second NPs injection followed by three additional US stimulations over consecutive days was included to enhance the combined treatment’s efficacy (Figure 4a). The most promising results were observed in the group treated with lipid-coated-IR780 NPs and US, as reflected by the significantly lower tumor weights compared to groups not receiving US (*p* < 0.007) (Figure 4b) and noticeable tumor shrinkage (Figure 4c). Tumor sizes, measured in vivo over 14 days, were consistent with ex vivo measurements and showed significantly slower tumor growth in the group treated with lipid-coated-IR780 NPs and US (Figure 4d). IVIS imaging confirmed that all treatment groups retained NPs in the tumors until study end point (Figure 4e). In particular, the fluorescence intensity of the nanoconstructs decreased over time until day 7 followed by restoration after the second NP administration (Figure 4f). Interestingly, the first US stimulation resulted in a slight increase in the total radiant efficiency across all US-treated groups (Figure 4g). No significant fluorescence intensity with respect to control groups was detected in other organs (kidneys, lymph nodes, spleen, liver, lungs, and scabs) despite the second NP administration except for a slight increase in groups treated with lipid-coated-IR780 NPs (Figure S6a–f). Finally, no spectral overlap with the signal of AlexaFluor 700-dyed NPs was detected at the excitation and emission wavelengths of the IR780, excluding any kind of fluorescence cross-talk (Figure S6g,h).

**Figure 5:**
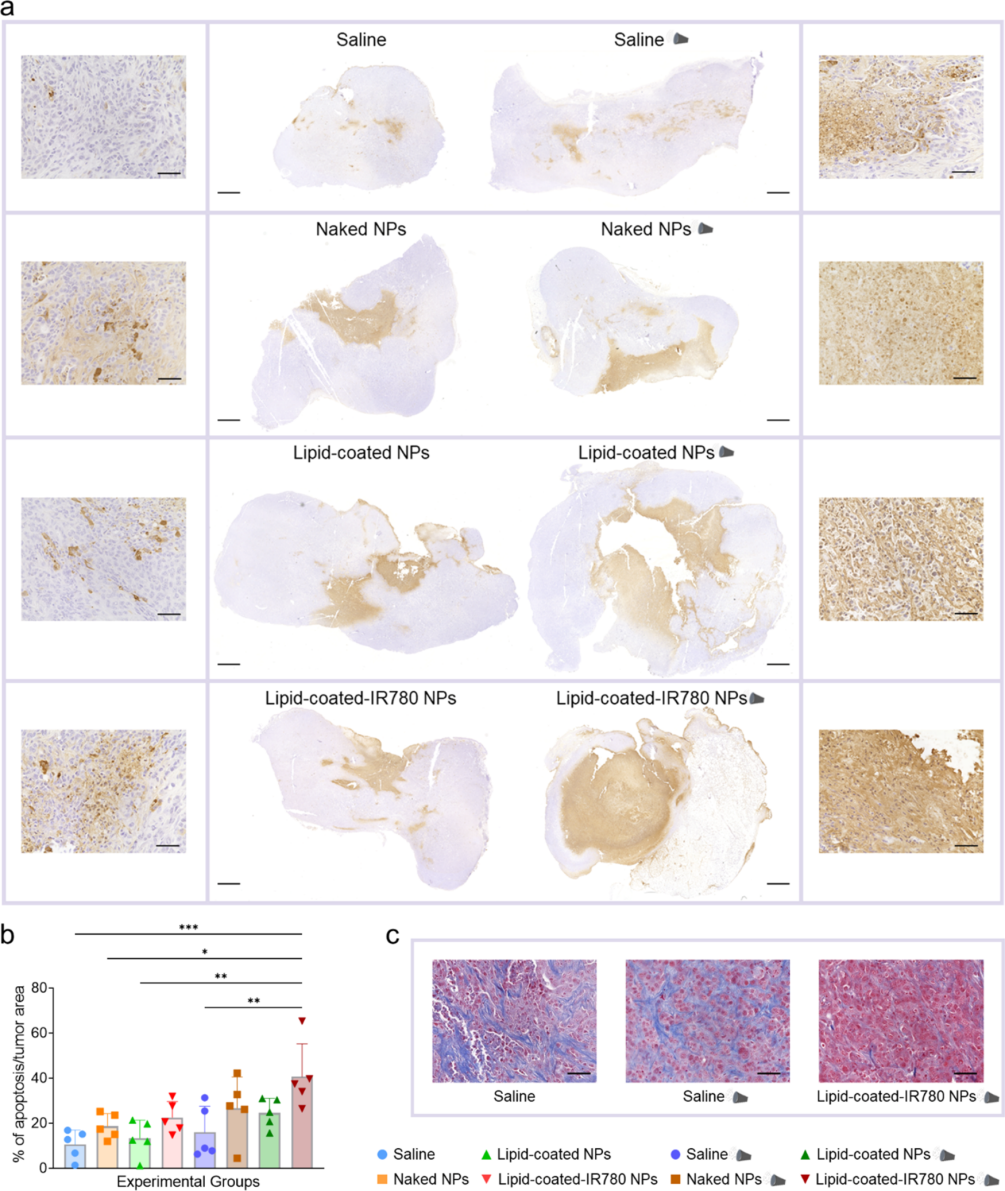
(a) Representative tumor sections, stained with the apoptosis assay, showing increasingly bigger apoptotic regions (brown) with respect to the total tumor slice area in groups receiving the combined treatment (scalebar 1 mm) and corresponding magnifications (scale bar 50 μm). (b) Percentage of apoptotic areas with respect to total tumor area for all treatment groups (*n* = 5/group). Data are expressed as mean ± standard deviation. Significance was analyzed by one-way ANOVA. *p < 0.05; **p < 0.005; ***p < 0.0005; ****p < 0.0001. (c) Representative Masson’s Trichrome staining of tumor slides where collagen fibers, stained in blue, decrease in the sample receiving the combined treatment (scalebar 50 μm).

Tumor slides subjected to TUNEL staining revealed an increase in the number of apoptotic cells in samples treated with both NPs and US, especially when the sonosensitizer was present in the lipidic shell (Figure 5a). These results underscored that the combination treatment induced significant apoptosis in pancreatic cancer cells, suggesting it as a potential primary route of cell death due to treatment. Quantification of the apoptotic area relative to the total tumor section area confirmed that the treatment consisting of lipid-coated-IR780 NPs and US led to statistically larger apoptotic regions (Figure 5b). Tumors treated with the combination of lipid-coated-IR780 NPs and US and stained with Masson’s Trichrome showed a reduction in fibrosis, resulting in a morphology more closely resembling healthy tissue.^47−49^ On the contrary, untreated tumors and tumors receiving US only revealed a predominance of collagen fibers, associated with the dense stroma characteristic of PDAC (Figure 5c).

### Tumor Immune Infiltration Assessed by Flow Cytometry

**Figure 6:**
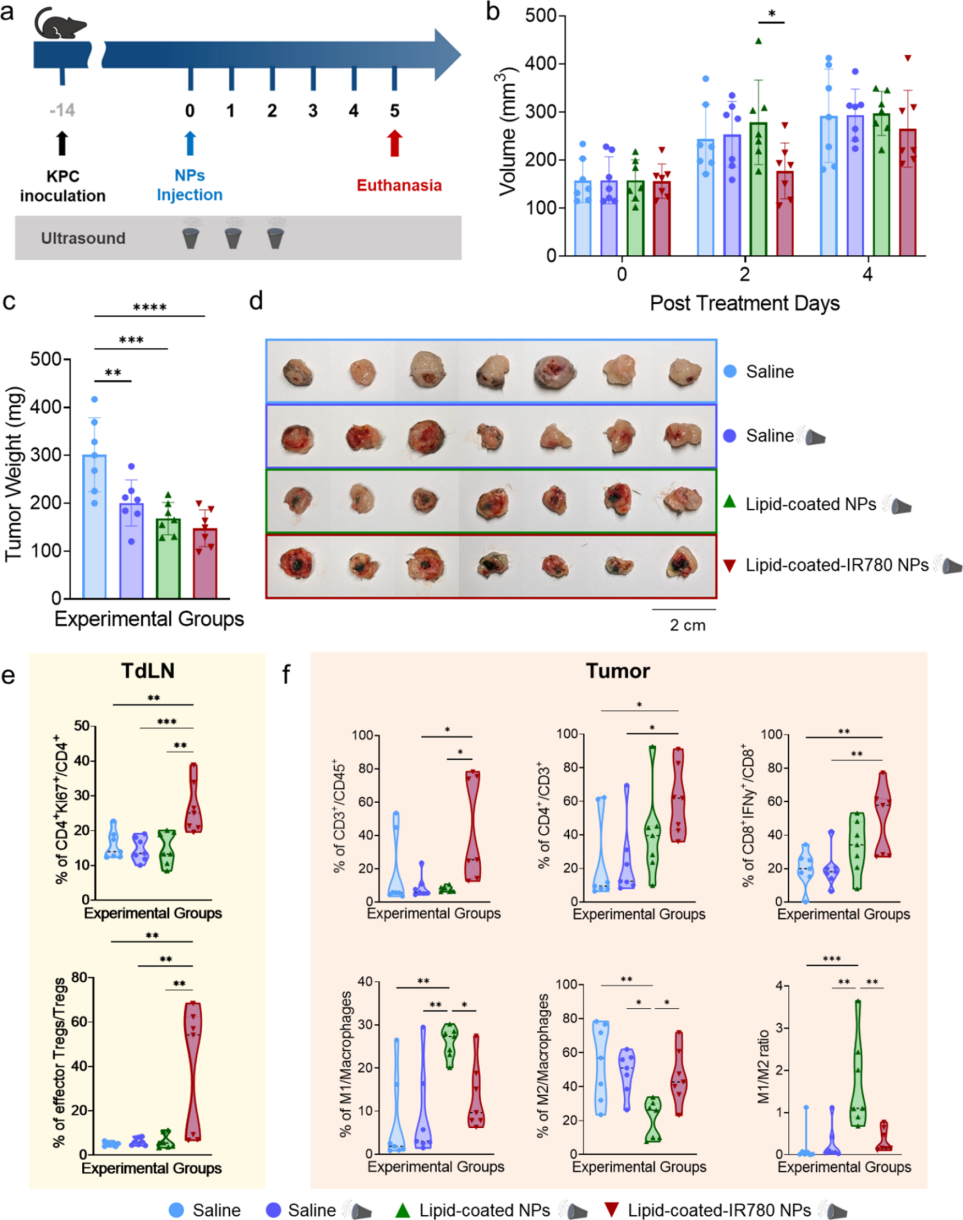
(a) Scheme of the treatment plan. (b) Tumor volume progression in vivo. (c) Tumor weights of the explanted tumors. (d) Digital photos of the explanted tumors. (e) Percentage of CD4^+^Ki67^+^ cells (top) and effector Tregs (CD4^+^CD25^+^Foxp3^+^CTLA-4^+^, bottom) in TdLN. (f) Percentage of CD3^+^, CD4^+^ and CD8^+^ki67^+^ cells (top) and M1, M2, and M1/M2 ratio (bottom) in tumors. Data are expressed as mean ± standard deviation. Significance was analyzed by either one-way or two-way ANOVA. *p < 0.05; **p < 0.005; ***p < 0.0005; ****p < 0.0001. Tukey’s correction was applied for multiple comparison.

Flow cytometry was used to assess immune infiltration in response to the combined therapy in a subset of treatment groups. Tumors were explanted on day 5, to prevent excessive shrinkage of those treated with lipid-coated-IR780 NPs, ensuring consistent comparisons among groups (Figure 6a). Although almost no significant difference in tumor volumes was detected in vivo over time (Figure 6b), tumor weights ex vivo indicated a significant reduction in treated groups compared to the saline control (Figure 6c), as further evidenced by their visible shrinkage (Figure 6d). To investigate the immune response due to the combined treatment, myeloid and lymphoid cell panels were tested on spleens, tumors, and tumor draining lymph nodes (TdLNs). No major systemic response was observed in the spleens, however interesting findings were noted in the TdLNs and tumors.

In the lymph nodes, an increase in proliferating helper T cells (CD4^+^Ki67^+^) suggested enhanced T cell activation, primarily supporting cytotoxic CD8^+^ T cells against tumor cells (Figure 6e). A statistically significant increase in effector Tregs (here defined as CD4^+^CD25^+^Foxp3^+^CTLA-4^+^ T cells) was observed in the experimental group treated with lipid-coated-IR780 NPs and US. Although Tregs are usually associated with poor prognosis, effector Tregs exhibit a more effector-like response and can help maintain immune balance.^50^ Their increase, indicating inflammation, could be due to the presence of ROS.

Tumors treated with lipid-coated-IR780 NPs and US showed an increase in infiltrating T cells (CD3^+^), associated with tumor cell detection and elimination. Specifically, there was a marked increase in CD4^+^CD3^+^ T cells, which can activate cytotoxic CD8^+^ T cells and whose presence is linked to improved survival and outcomes. Additionally, a marked increase of CD8^+^ T cells producing interferon-γ (IFN-γ^+^CD8^+^), a cytokine involved in the activation and proliferation of antitumor CD8^+^ T cells, was observed, with important implications for tumor cell apoptosis and elimination (Figure 6f).

Strikingly, this experimental group did not show an increased polarization toward M1-like macrophages, contrary to previous expectations based on the in vivo efficacy study, where tumor reduction was observed. On the other hand, the group treated with US but lacking the sonosensitizers in the lipidic shell showed an increase in M1-like macrophages (CD80^+^F4/80^+^CD11b^+^), a decrease in M2-like macrophages (CD206^+^F4/80^+^CD11b^+^), and an increased M1/M2 ratio, demonstrating an antitumoral response due to the treatment. A possible explanation for these observations could be a complex interplay between treatment-dependent ROS generation and the immune cell response. Excessive ROS production might disrupt the balance between M1 and M2 macrophage polarization and impair antigen presentation.^51,52^ Preliminary data (Figure 1i) indicated that ROS production was significantly higher with the lipid-coated-IR780 NPs than without the sonosensitizer, supporting this hypothesis.

Additionally, a potential limitation related to the experimental design of flow analysis must be acknowledged. By restricting the immune cell phenotypes characterization to a single time point, we potentially missed time-dependent events and dynamic changes occurring before or after day 5. Despite this potential limitation, there were measurable differences in lymphoid and myeloid cells, with marked statistical significances between groups treated with lipid-coated NPs (either with and without IR780) and US vs saline + US. Notably, US stimulation alone did not affect M1 polarization nor enhance cytotoxic T cell infiltration in the tumor area, suggesting that the presence of NPs is required to initiate this whole chain of events leading to local immune response.

### Survival Study on Mice Receiving the Combined Therapy

**Figure 7:**
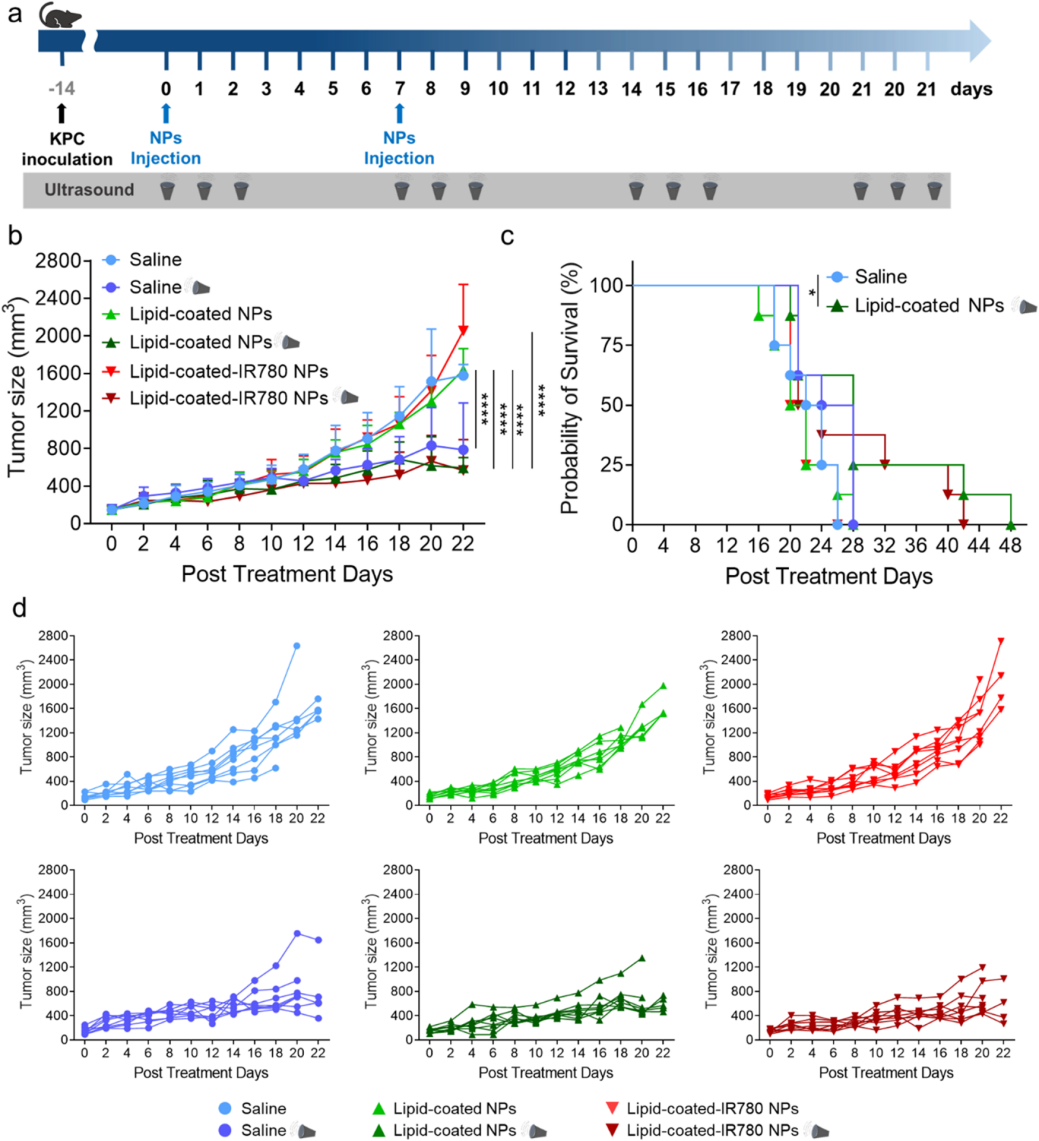
(a) Scheme of the treatment plan. (b) Tumor volume progression in vivo. Data are expressed as mean ± standard deviation. Significance was analyzed by two-way ANOVA. *p < 0.05; **p < 0.005; ***p < 0.0005; ****p < 0.0001. Tukey’s correction was applied for multiple comparison. (c) Kaplan–Meier survival curves. Significance was analyzed by log-rank test; *n* = 8/group; **p < 0.001; ***p < 0.0005. (d) Tumor growth curve in vivo, each plot referred to a single experimental group.

To evaluate the long-term efficacy of the treatment, mice were treated for 2 successive weeks with an injection of NPs followed by three daily consecutive US irradiations and then treated weekly with US only, until the humane end points were reached (Figure 7a). Tumor volume progression in vivo showed a clear and consistent response to the treatment, dependent on the presence of US stimulation in the treatment groups (Figure 7b,d).

Mice receiving only NPs quickly reached the tumor volume end point, with a median survival of 21 days. In contrast, the mice undergoing the combined treatment exhibited significantly prolonged survival, with median survival of 28 and 22.5 days, lasting up to 48 days after the first injection. The mice receiving only US stimulation died within 28 days from the first treatment, due to tumor cavitation and ulceration (Figure 7c).

## Conclusions

This study demonstrates the potential of US-stimulated zinc oxide NPs as an alternative therapeutic approach for pancreatic cancer. Our nanoconstructs are consistently reproducible and are thoroughly characterized, exhibiting safety and biocompatibility due to the protective lipidic shell. In vitro data show that ROS generation is triggered by US application on the NPs, leading to tumor cell death. In vivo, the combined therapy results in tumor shrinkage, likely due to cancer cell apoptosis, significant immune cell infiltration with macrophage polarization, and prolonged mouse survival.

Although a degree of antitumor effect in KPC mice is observed with US treatment alone, flow cytometry data indicate that only the combination of lipid-coated(-IR780) NPs and US could be advantageous for generating an antitumor immune response.

While the combined therapy did not achieve complete tumor clearance in vivo, an essential consideration is the inherent safety of the individual treatment components. The injected NPs were far below any subtoxic threshold, and the US samples were administered through an FDA-approved transducer, allowing for numerous future potential combinations by simply tuning NPs dosages and treatment durations.

The versatile lipidic shell inspired by COVID-19 vaccine formulations allows for the potential inclusion of various functional lipids, covalently bound to targeting peptides,^28^ antibodies,^41^ or proteins. Thus, combining this treatment with immunotherapy, such as incorporating an agonistic anti-CD40 monoclonal antibody covalently bound to the lipidic shell, could further enhance the systemic immune response, leveraging the multimodal therapy potential of the nanoconstructs. Therefore, minor adjustments in the nanoconstruct design and dosage, along with intravenous injections, could be implemented in future experiments toward a more realistic clinical setting.

Based on the results, we postulate that a stronger efficacy in the reduction of tumor growth may be attained with higher NP concentrations. Furthermore, it is possible that other cancer models may be more responsive than the highly fibrotic and desmoplastic KPC model. Additionally, the results suggest that the effect of NPs on tumor reduction may be more visible at a later stage, necessitating further investigations.

Overall, the study confirms that the combination of NPs and US could effectively treat aggressive tumors, such as pancreatic cancer. These findings provide a strong foundation for future research into innovative multimodal therapies for pancreatic cancer.

## Experimental Section

### Nanoconstructs Fabrication and Characterizations

Oleic acid capped and iron-doped zinc oxide NPs were synthesized following a protocol developed by Carofiglio et al.^26^ and characterized by dynamic light scattering (DLS), ζ-potential, nanoparticle tracking analysis (NTA), scanning electron microscopy (SEM), energy-dispersive X-ray (EDX), and X-ray diffraction (XRD) analyses.

The lipidic shell formulation used to coat the NPs consisted of DOPA (18:1 PA, 1,2-dioleoyl-*sn*-glycero-3-phosphate (sodium salt), chloroform solution), DOPC (18:1 (Δ9-cis) PC (DOPC), 1,2-dioleoyl-*sn*-glycero-3-phosphocholine, chloroform solution), and DSPE-PEG(2000) amine (1,2-distearoyl-*sn*-glycero-3-phosphoethanolamine-*N*-[amino(polyethylene glycol)-2000] (ammonium salt)) and cholesterol solution in chloroform, purchased by Avanti Polar Lipids Inc. (Birmingham, AL). The coating was achieved using a simple solvent exchange method, as recently described.^28^ To assess the efficacy of the lipidic coating, DLS, ζ-potential, and NTA and Cryo-EM analyses were carried out.

For CryoEM imaging, naked and lipid-coated NPs were vitrified and imaged at the Baylor College of Medicine Cryo-Electron Microscopy Core Facility (Texas Medical Center, Houston). A Pelco EasiGlow machine (Ted Pella, Inc.) was used to glow discharge Quantifoil R1.2/1.3 300Cu (Quantifoil Micro Tools GmbH, Jena, Germany). Each grid was transferred to a Vitrobot Mark IV (FEI Company, Hillsboro, OR) where 3 μL of the sample was applied to the grid. The samples were blotted and vitrified, and then the grids were transferred to a Thermo Fisher Glacios Electron Microscope (Thermo Fischer Scientific, Inc.) operating at 200 kV. Images were captured using the built-in EPU and Velox programs.

A lipophilic sonosensitizer, IR780 (Sigma-Aldrich), was incorporated into the lipidic shell and dried together with the lipids. A calibration curve of IR780 in water was employed to calculate IR780 retention in the lipidic shell, which was analyzed with UV–vis spectroscopy after centrifugation of lipid-coated-IR780 NPs (Figure S2a–d).

To concentrate the NPs in saline solution in view of in vivo applications, the following protocol was developed (Figure S2e). NPs were centrifuged, redispersed in a small volume of water, and then combined with a 10× concentrated saline solution to achieve a final 0.9% w/v saline solution without impairing the lipid coating.

NPs were labeled with either AlexaFluor 647 or AlexaFluor 700 for in vitro flow cytometry and fluorescence microscopy studies as well as for in vivo IVIS imaging studies (Figure S2g–i).

### ROS Production in Water

To evaluate ROS production under US stimulation, EPR coupled to the spin trapping technique was performed. A water solution containing a hydroxyl radical chemical trap 5,5-dimethyl-l-pyrroline-*N*-oxide (DMPO, Sigma-Aldrich) and the nanoconstructs was prepared. The solution was stimulated with US radiation at different power densities (0.2–2.0 W/cm^2^), at 1 MHz for 1 min, with a 2 cm^2^ US transducer (Chattanooga Intelect Transport Ultrasound, DJO LLC) and coupled with an acoustic water-based gel (Stosswellen Gel, ELvation Medical GmbH). The hydroxyl radical concentration was immediately measured using an EMXNano X-band spectrometer (Bruker, center field 3426 G, 10 scans, 60 s sweep time), and data were processed using the Bruker Xenon software (Bruker).

### ICP-OES

An Agilent 5800 ICP-OES machine was used to assess the dissolution of zinc in either saline solution or cell culture medium, using Yttrium (Sigma-Aldrich, St. Louis, MO, Cat#01357) as an internal standard. Calibration curves were obtained using a Zinc standard for ICP (TraceCERT, 1 g/L Zn in nitric acid) opportunely diluted. Wavelengths of 202.548 and 213.857 nm were used to measure zinc emission. The zinc concentration was obtained using ICP-OES software ICP Expert (v7.6) and averaged between the two wavelengths. Briefly, 200 μg of naked or lipid-coated NPs was dispersed in 200 μL of saline or cell culture medium in Eppendorf tubes, and they were stirred at 37 °C for up to 1 week. At different time points (1, 5, 24, 72, and 168 h), the samples were centrifuged, and both the pellet and the supernatant were digested in 1.8 mL of aqua regia (a mixture of nitric acid and hydrochloric acid, in a molar ratio of 1:3). All samples were diluted with 2 mL of a solution composed of 10% hydrochloric acid and 1% nitric acid (diluting solution) and analyzed. All experiments were conducted in triplicate, and the sum of the amount of zinc detected in the pellet and supernatant was set to 100% for each sample.

### Cytotoxicity

The cytotoxicity of the nanoconstructs was preliminarily tested on KPC cells (gifted by Sankar Mitra, Houston Methodist Research Institute). Different concentrations of naked NPs, lipid-coated NPs, and lipid-coated NPs containing the IR780 sonosensitizer were added directly into the culture media (Gibco medium DMEM/F12 supplemented with 10% of fetal bovine serum, ATTC, 1% of 100 μg/mL of streptomycin, and 100 units/mL of penicillin, Sigma-Aldrich) and administered to cells, and their viability was determined using WST-1 (Roche) assay after 24, 48, and 72 h of incubation.

### Ultrasound Efficacy Study

The efficacy of US treatment on KPC cells seeded in 24-well plates was evaluated with an ultrasound transducer (Chattanooga Intelect Transport Ultrasound, DJO LLC). Cells were treated with US 24 h after NP administration. Cells were treated for 1 min, with a power density of 0.8 and 1 W/cm^2^, at a frequency of 1 MHz and a continuous operation mode (DC 100%) with the 2 cm^2^ applicator, coupled with a water-based gel; then, they were detached and seeded in 96-well plates to perform the viability assay after further 24 and 48 h at standard cell culture conditions.

### Internalization Study

To assess NPs internalization, a protocol by Giordano et al.^53^ was carried out as follows: cells were seeded in 24-well plates (μ-Plate 24 Well ibiTreat, ibidi) and treated with lipid-coated-IR780 NPs, where ZnO was previously labeled with AlexaFluor 647 NHS ester. After 24 h, the ultrasound treatment was carried out with a power density of 0.8 W/cm^2^. Then, cells were incubated with ROS probe, 2′,7′-dichlorofluorescein diacetate (DCF-DA) (Invitrogen), at standard cell culture conditions for 30 min in PBS, following a protocol by Liu et al.^54^ DCF-DA is a cell-permeable nonfluorescent probe that becomes fluorescent in the presence of intracellular ROS, by oxidation to dichlorofluorescein (DCF).

Then, the cells were washed with PBS, fixed with paraformaldehyde, 2% in PBS for 10 min at room temperature, washed twice, and incubated with 5 μg/mL wheat germ agglutinin (WGA) S-10 AlexaFluor 550 Conjugate (Invitrogen) for 10 min in standard cell culture conditions, then washed twice again with PBS and incubated with 1 μg/mL DAPI (Abcam) for 3 min at room temperature. After a last wash, walls were removed, a drop of ProLong Diamond Antifade Mountant media (Invitrogen) was placed on the slide, and a coverslip was placed on top. Images were acquired with a confocal fluorescence microscope (Olympus FV3000).

### Flow Cytometry Study

Cells were seeded in 24-well plates, treated with a safe dose of all treatment groups of NPs (previously labeled with AlexaFluor 647 NHS ester, Invitrogen), and received ultrasound stimulation (1 min, 0.8 W/cm^2^). Right after the stimulation, cells were trypsinized and centrifuged, media was replaced with PBS and DCF-DA was added and incubated at standard cell culture conditions for 30 min. DAPI was then added for nuclei staining and the obtained cell suspension was analyzed with a BD FACSymphony A5 SE Cell Analyzer (BD Biosciences). Events were acquired employing the R660 channel to detect AlexaFluor 647-labeled NPs, the R780 channel to detect the signal of IR780, the B510 channel to detect the ROS probe, and the UV446 channel to gate viable cells.

### Animals

6-Week-old C57BL/6 mice (6 weeks old) were purchased from Taconic Biosciences (Rensselaer, New York). The mice were kept in the comparative medicine facility at the Houston Methodist Research Institute (HMRI, Houston, TX). They underwent a 72 h acclimation period to their new environment and received unrestricted access to food and water on a 12 h day/night cycle. All experiments adhered to protocols reviewed by an independent Institutional Animal Care and Use Committee (IACUC), protocol no. IS00007365, and following the guidelines of the National Institutes of Health Guide for the Care and Use of Laboratory Animals and Animal Welfare Act and the ARRIVE guidelines.

### Tumor Model

To establish the PDAC tumor model, 1 × 10^6^ KPC cells were suspended in 100 μL of a 3:1 mixture of PBS and Matrigel (Corning, CB40234) and inoculated subcutaneously in the right flank. Mouse KPC cell line was chosen since it models the disease accurately, retaining key genetic mutations and providing a well-established tumor model for PDAC.^55−57^ Tumor volume, mice weight, and basal temperature were monitored every other day with a digital caliper and a scale. Tumor volume was determined using the formula (length × width^2^)/2, where length represents the longest dimension and width is its perpendicular counterpart.

### Biodistribution Study

When tumors reached 100–150 mm^3^ in volume, mice were randomized into designated groups (*n* = 5/group) and treated with naked and lipid-coated NPs (previously labeled with AlexaFluor 700 NHS ester (Invitrogen)), dispersed in saline solution, and injected intratumorally at a dose of 30 mg/kg of weight. Control mice received saline solution only. The mice were monitored for 14 days after the injection and imaged with an in vivo bioluminescence/fluorescence imaging system (IVIS Spectrum, PerkinElmer). The right flank was shaved before acquiring the baseline image, and mice anesthetized with isoflurane were fluorescently imaged to detect the signal of NPs at specific time points, (excitation of 675 nm and emission of 720 nm). Two small cohorts of *n* = 4 animals per group were also treated with NPs and sacrificed after 1 and 3 days from NPs injection. At the established end points, all animals were euthanized by CO_2_ asphyxiation and organs were harvested for ex vivo imaging. The images were processed by Living Image Software (PerkinElmer) by drawing a region of interest (ROI) around the tumor area in vivo and the extracted organs, measuring the fluorescent signal in radiance ([p/s]/[μW/cm^2^]). Blood was collected in BD Microtainer collection tubes, centrifuged at 5000*g* for 12 min, and the obtained plasma was frozen at −80 °C for further analyses, together with the harvested tumors, previously weighed.

### ICP-OES of Tumors and Plasma

Tumors and plasma were dissolved in aqua regia by adapting an established digestion protocol.^58,59^ Briefly, tumors were thawed and immersed in 4 mL of fresh aqua regia overnight under a chemical fume hood on a heating plate (*T* = 60 °C). After complete digestion, 8 mL of diluting solution was added, and the final volume was filtered with 0.45 μm pore size Nylon syringe strainers (Whatman Puradisc). Plasma was thawed, and 200 μL from each sample was digested in 2 mL of aqua regia overnight under a chemical fume hood. Afterward, 4 mL of diluting solution was added to the digested samples, and a filtration step was performed before the analysis. All measurements were performed in triplicate.

### Ultrasound Efficacy Study In Vivo

After reaching a tumor volume of 100–150 mm^3^, 6 mice per group were treated with naked, lipid-coated, and lipid-coated IR780 NPs, as described above. Control mice received saline solution. The mice were anesthetized and shaved in the tumor area, and a baseline image was acquired with IVIS Spectrum. To detect the signal of IR780, the excitation wavelength was set at 745 nm and the emission at 820 nm. Right after the injection, another IVIS image was acquired and the US stimulation (2 W/cm^2^, 1 MHz, DC 100%, 2 min) was applied with the 2 cm^2^ applicator. Water-based gel was employed to avoid any unwanted overheating. The mice were imaged once again with the IVIS Spectrum. The US treatment was repeated for the 2 following days, and IVIS pictures were collected at established time points. After 7 days from the first NP administration, the treatment was repeated with a second injection of NPs at the same dose and a US treatment for 3 consecutive days. After 14 days from the first injection, the mice were sacrificed. Tumors and organs were collected and imaged with IVIS and formalin-fixed.

### Histopathology Analysis of Tumors

Tumors were harvested, imaged with IVIS, and then formalin-fixed, paraffin-embedded (FFPE), and stained (*n* = 5/group) with an apoptosis kit (ApopTag Peroxidase in Situ Apoptosis Detection Kit, Sigma-Aldrich) and Masson’s Trichrome. Ten ROI from each section and the full tumor area were acquired with an inverted fluorescence microscope (Keyence BZ-X810) and analyzed with Keyence BZ-X800 Analysis Software. ImageJ software was employed to apply color deconvolution and calculate the percentage of the apoptotic area with respect to the total tumor area in *n* = 5/group samples stained with ApopTag.

### Flow Cytometry Study

Saline solution, lipid-coated NPs, or lipid-coated-IR780 NPs were intratumorally injected, and US stimulation was applied for 3 consecutive days of all groups (*n* = 6/group), as previously described. Mice receiving only saline solution and no US treatment were set as the control. After 5 days from the NPs administration, all mice were sacrificed, and tumors, TdLNs, and spleens were collected and processed for flow cytometry. Briefly, spleens were dissociated into single-cell suspensions by mechanical filtration through 40 μm cell strainers (Fisherbrand, 22-363-547) and red blood cells were lysed with two cycles of ACK lysis buffer (Quality biological, 118-156-101). TdLNs were digested in RPMI-1640 with 1× collagenase/hyaluronidase (STEMCELL Technologies, NC2031808) and mechanically dissociated through 40 μm cell strainers. Tumors were cut into 1 mm sections and then incubated for 1 h at 37 °C on an orbital shaker in RPMI-1640 with 1× collagenase/hyaluronidase and 20U mL^–1^ of DNase I (Sigma-Aldrich, 11284932001), then mechanically dissociated through 40 μm cell strainers into single-cell suspension. Tumor-infiltrating leukocytes were then separated using Lymphoprep (StemCell Technologies, NC0665098). Cells from all of the organs were finally resuspended in FACS buffer (Corning Dulbecco’s Phosphate-Buffered Saline, 1× without calcium and magnesium, 21-031-CV + 2% of Corning Fetal Bovine Serum, 35-011-CV) and plated in 96-well V-bottom plates. Cells were washed and stained with Rat Antimouse CD16/CD32 (BD Biosciences, 553141) for 30 min, then stained with either the myeloid or lymphoid antibody panel. Events were acquired employing the A5 SE FACSymphony Cell Analyzer (BD Biosciences) and analyzed using FlowJo v10.9 software.

### Survival Study

A survival study was conducted on a separate mouse cohort (*n* = 8/group), following the same treatment plan described in the US Treatment section. Additionally, the US irradiation was repeated every week for 3 consecutive days until the mice reached the humane end point; the tumor mass was measured, along with the body weight and rectal temperature, every other day. Kaplan–Meier survival curves were generated, and log-rank tests were used to compare the survival between groups.

### Statistical Analysis

Statistical analyses were conducted using GraphPad Prism 9.1.6. An analysis of variance (ANOVA) was employed for multiple group comparisons. Significance was analyzed by either unpaired one-way or two-way ANOVA. Multiple comparisons were carried out with Tukey‘s correction. *p* < 0.05 was considered statistically significant: *p < 0.05; **p < 0.005; ***p < 0.0005; ****p < 0.0001.

## Supporting Information Available

Additional experimental details on zinc release in saline and cell culture medium; lipid-drying protocol incorporating the IR780 sonosensitizer and related evidence; cytotoxicity experiments on KPC cells; animal experiments additional data, like tumor volume progression, photos of tumors explanted on day 14, mice weight and temperature progression over time; total radiant efficiency of explanted organs and tumors over time and at day 14 (PDF)
