# Supplementary material for: Acoustically Driven Hybrid Nanocrystals for In Vivo Pancreatic Cancer Treatment
Source: ACS Appl Mater Interfaces. 2025 Feb 17;17(8):11873–87. doi: 10.1021/acsami.4c21975 (PMC11873934; doi:10.1021/acsami.4c21975)
Supplement: Supplementary file 1 — am4c21975_si_001.pdf [file am4c21975_si_001.pdf]

Supporting Information

Acoustically-driven Hybrid Nanocrystals for In Vivo Pancreatic Cancer Treatment

Marzia Conte[†‡], Marco Carofiglio[†], Robin Shae Vander Pol[‡], Anthony Wood[‡], Nathanael Hernandez[‡ǂ], Ashley Joubert[‡], Camden Caffey[‡], Corrine Ying Xuan Chua[‡], Alessandro Grattoni[‡¥#] and Valentina Cauda[†*]*

[†] Department of Applied Science and Technology, Politecnico di Torino, Corso Duca degli Abruzzi 24, 10129, Turin, Italy

[‡] Department of Nanomedicine, Houston Methodist Research Institute, Houston, TX 77030, USA [¥] Department of Surgery, Houston Methodist Research Institute, Houston, TX 77030, USA

[#] Department of Radiation Oncology, Houston Methodist Research Institute, Houston, TX 77030, USA

[ǂ] Deceased September 16th, 2024

* Corresponding Authors:

Prof. Valentina Cauda, Phone: +39 011 090 7389, e-mail: valentina.cauda@polito.it

Prof. Alessandro Grattoni, phone: +1 832 667 5916, e-mail: agrattoni@houstonmethodist.org

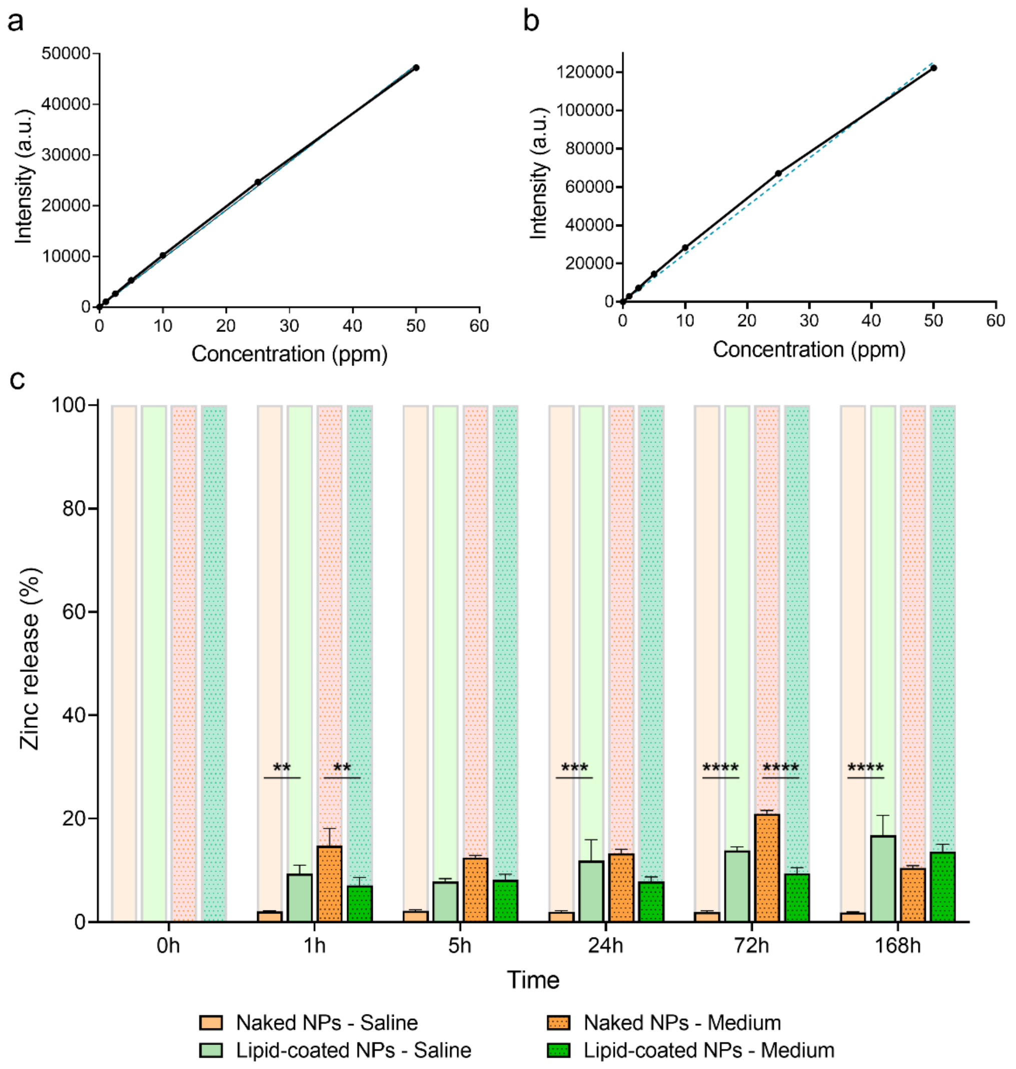

Figure S1. a) Zinc standard calibration curves obtained at 202.548 nm and b) 213.857 nm. c) Histograms reporting zinc release in saline and cell culture medium (patterned columns) of naked (orange) and lipid-coated (green) NPs over one week of incubation at 37 °C. Data are expressed as mean ± standard deviation. Significance was analyzed by two-way ANOVA. *$p < 0.05$; **$p < 0.005$; ***$p < 0.0005$; **** $p < 0.0001$. Tukey's correction was applied for multiple comparison.

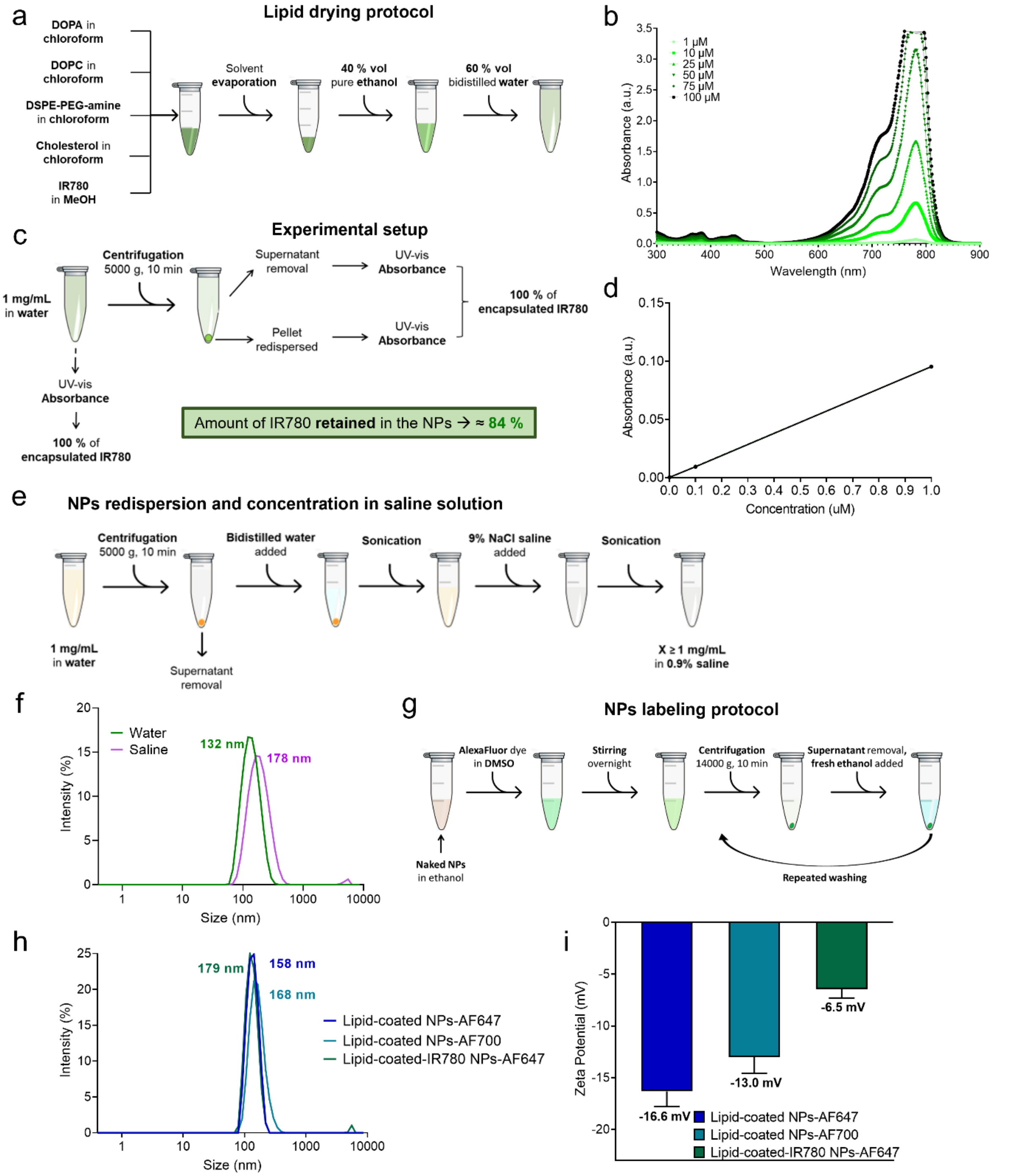

Figure S2. a) Scheme of the lipid-drying protocol incorporating the IR780 sonosensitizer. b) IR780 absorbance spectrum in methanol at different concentrations. c) Experimental setup to assess IR780 retention in the lipidic shell. d) Calibration curve of IR780 in water employed to evaluate IR780 retention. e) Protocol to resuspend NPs in saline solution at higher concentrations than in water (up to 40 mg/mL), consisting in a first redispersion in a small volume of water and

the successive addition of 10x concentrated saline solution, to get to a 0.9 %v/w saline solution suitable for injection. f) DLS of lipid-coated NPs in water (green) and saline solution (purple). g) NPs labeling protocol with fluorescent dyes for imaging, flow cytometry and in vivo applications. h) DLS and i) zeta potential of lipid-coated-AlexaFluor647 (blue), lipid-coated-AlexaFluor700 (cyan) and lipid-coated-IR780-AlexaFluor647 NPs (dark green). The observed shift in zeta potential towards negative values is due to the dye coupling, covalently bound to amino-propyl groups on the NP surface. Data are expressed as mean ± standard deviation.

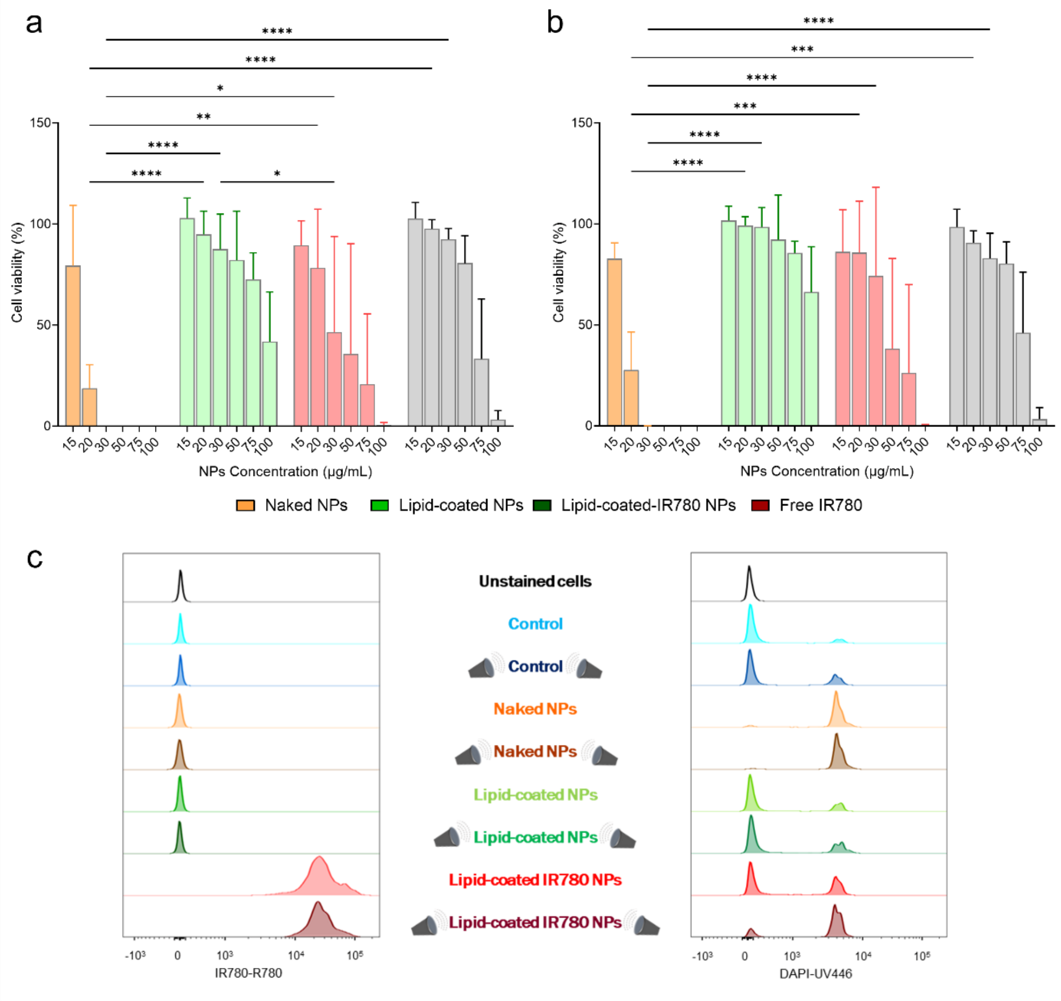

Figure S3. Cytotoxicity on KPC cells after a) 48h and b) 72h from NPs administration. Data are expressed as mean ± standard deviation. Significance was analyzed by two-way ANOVA. *$p < 0.05$; **$p < 0.005$; ***$p < 0.0005$; **** $p < 0.0001$. Tukey's correction was applied for multiple comparison. c) One-dimensional histograms reporting the fluorescent intensity associated with cells internalizing IR780 (IR780-R780) and dead cells positive to DAPI (DAPI-UV446).

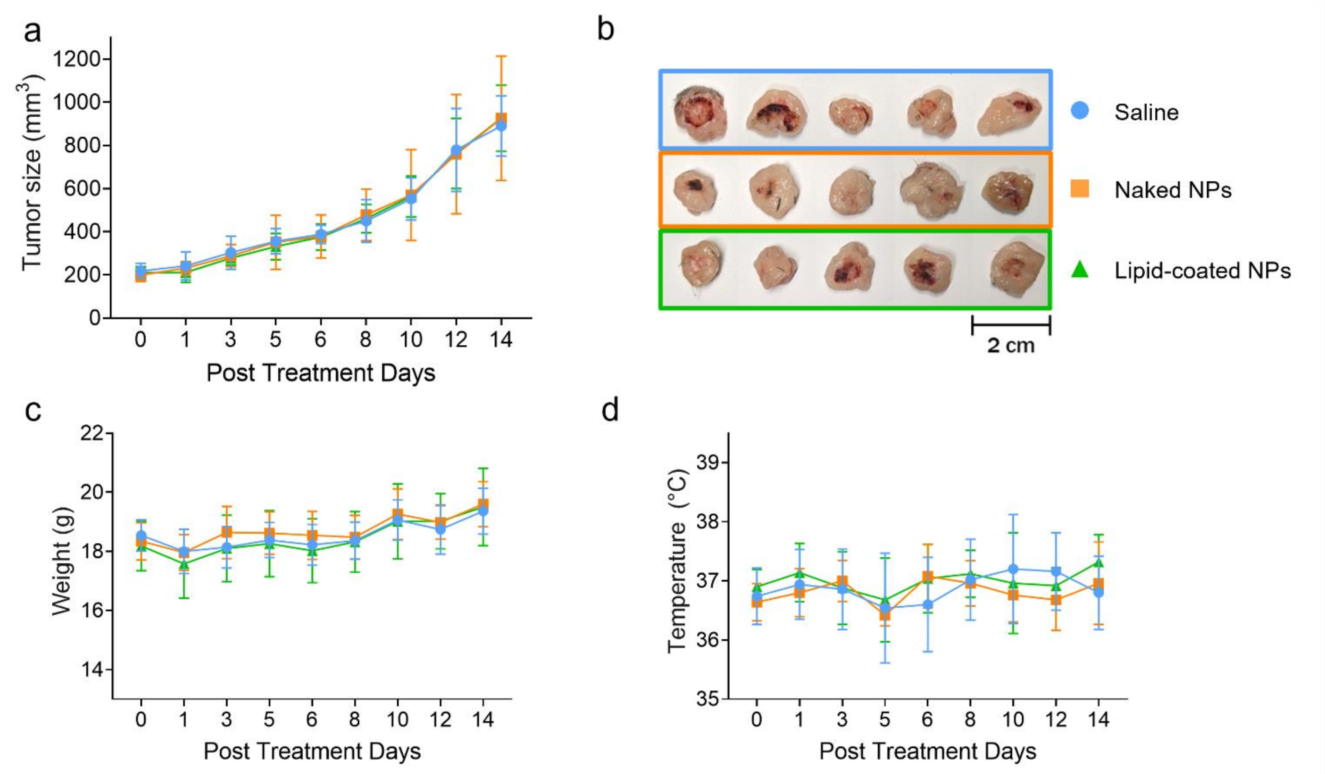

Figure S4. a) Tumor volume progression in vivo. b) Digital photos of tumors explanted on day 14. c) Mouse weight progression over time. d) Animal temperature progression over time. Data are expressed as mean ± standard deviation.

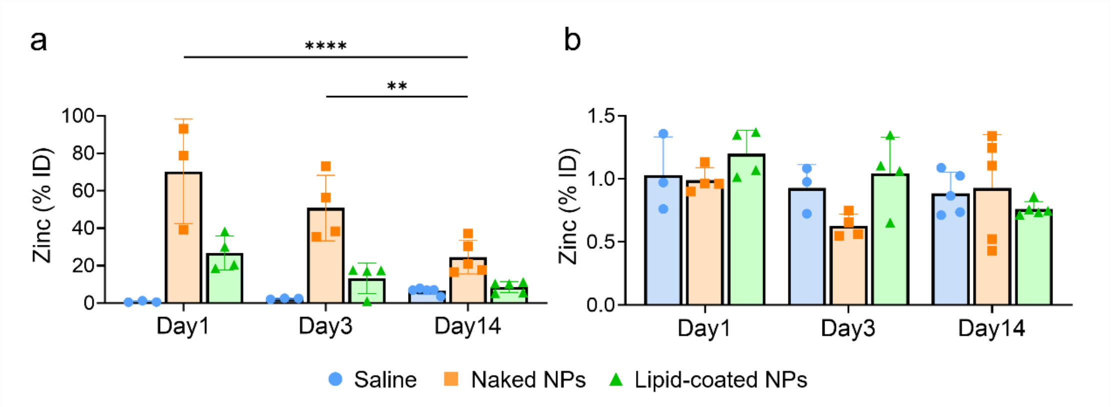

Figure S5. a) Zinc concentration expressed as a percentage of the injected dose in tumors and b) plasma. Data are expressed as mean ± standard deviation. Significance was analyzed by two-way ANOVA. *$p < 0.05$; **$p < 0.005$; ***$p < 0.0005$; **** $p < 0.0001$. Tukey's correction was applied for multiple comparison.

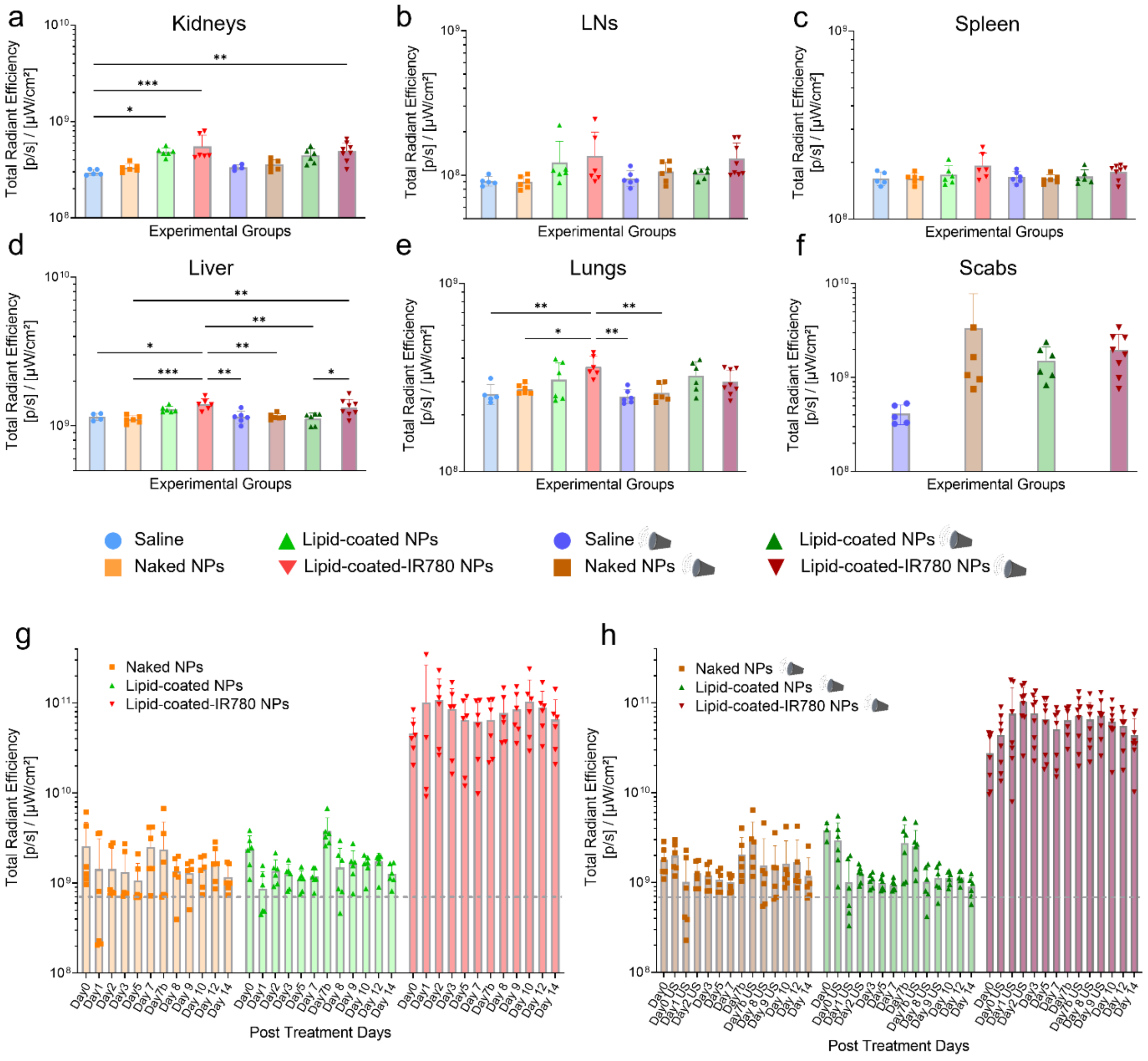

Figure S6. a-f) Histograms reporting the total radiant efficiency (excitation 675 nm, emission 720 nm) of kidneys, LNs, spleen, liver, lungs and scabs explanted on day 14. g) Histograms reporting the total radiant efficiency progression in vivo in groups treated with NPs only and h) groups treated with NPs and ultrasound stimulation (excitation 745 nm, emission 820 nm) to prove the absence of spectral overlap of AlexaFluor700 on the IR780 signal.
